# A comparative analysis of the distribution of immunoreactive orexin A and B in the brains of nocturnal and diurnal rodents

**DOI:** 10.1186/1744-9081-3-28

**Published:** 2007-06-13

**Authors:** Joshua P Nixon, Laura Smale

**Affiliations:** 1 Department of Zoology, Michigan State University, 203 Natural Science Building, East Lansing, MI 48824-1115 USA; 2 Department of Food Science and Nutrition and Minnesota Craniofacial Research Training Program (MinnCResT), 17-164 Moos Tower, 515 Delaware St. SE, Minneapolis, MN 55455-0357 USA

## Abstract

**Background:**

The orexins (hypocretins) are a family of peptides found primarily in neurons in the lateral hypothalamus. Although the orexinergic system is generally thought to be the same across species, the orexins are involved in behaviors which show considerable interspecific variability. There are few direct cross-species comparisons of the distributions of cells and fibers containing these peptides. Here, we addressed the possibility that there might be important species differences by systematically examining and directly comparing the distribution of orexinergic neurons and fibers within the forebrains of species with very different patterns of sleep-wake behavior.

**Methods:**

We compared the distribution of orexin-immunoreactive cell bodies and fibers in two nocturnal species (the lab rat, *Rattus norvegicus *and the golden hamster, *Mesocricetus auratus*) and two diurnal species (the Nile grass rat, *Arvicanthis niloticus *and the degu, *Octodon degus*). For each species, tissue from the olfactory bulbs through the brainstem was processed for immunoreactivity for orexin A and orexin B (hypocretin-1 and -2). The distribution of orexin-positive cells was noted for each species. Orexin fiber distribution and density was recorded and analyzed using a principal components factor analysis to aid in evaluating potential species differences.

**Results:**

Orexin-positive cells were observed in the lateral hypothalamic area of each species, though there were differences with respect to distribution within this region. In addition, cells positive for orexin A but not orexin B were observed in the paraventricular nucleus of the lab rat and grass rat, and in the supraoptic nucleus of the lab rat, grass rat and hamster. Although the overall distributions of orexin A and B fibers were similar in the four species, some striking differences were noted, especially in the lateral mammillary nucleus, ventromedial hypothalamic nucleus and flocculus.

**Conclusion:**

The orexin cell and fiber distributions observed in this study were largely consistent with those described in previous studies. However, the present study shows significant species differences in the distribution of orexin cell bodies and in the density of orexin-IR fibers in some regions. Finally, we note previously undescribed populations of orexin-positive neurons outside the lateral hypothalamus in three of the four species examined.

## Background

The orexins (hypocretins) are a recently described family of peptides originating in cells of the lateral hypothalamus [1,2]. Orexins are thought to be primarily involved in the regulation of arousal and sleep-wake behavior, general activity, body temperature, drinking, and feeding [3-18]. Anatomical studies of orexin fiber distribution in the rat brain show that the densest projections extend to the locus coeruleus, raphé nuclei, periaqueductal central gray, paraventricular hypothalamic nucleus, arcuate nucleus, and the lining of the third ventricle [19-24]. Orexin cell bodies in the rat are primarily limited to the perifornical nucleus and lateral hypothalamic area, with more sparse distributions in the dorsal hypothalamic area, posterior hypothalamic area and the dorsomedial hypothalamic nuclei [19-21,24]. Published descriptions of orexin cell and fiber distributions are generally similar to those in the rat for the Syrian and Djungarian hamster [25-27] as well as for humans [28-30].

The orexins consist of two peptides, orexin A (OXA, hypocretin-1) and orexin B (OXB, hypocretin-2), derived from the same precursor protein, preproorexin [1,2]. The orexins bind to two G-coupled protein receptors, orexin receptors 1 (OX_1_R, HCRTR-1) and 2 (OX_2_R, HCRTR-2) [1]. Although OXA and OXB appear to be equally effective in activation of OX_2_R, OXA is 30- to 100-fold more effective than OXB in activating OX_1_R [1,31]. The two orexin receptors exhibit distinctly different distribution patterns in the rat brain [reviewed in [32]]. For example, while the raphé nuclei, thalamus, and layer 6 of the cortex express OX_1_R and OX_2_R equally, only OX_1_R is present in cortical layer 5, hippocampal field CA1, and locus coeruleus (LC), whereas cortical layer 2, hippocampal field CA3, septal nuclei, and tuberomammillary nuclei express only OX_2_R [33-35]. The differential distribution and potential selectivity of the two orexin receptors raises the possibility that there may be some differences in the functional roles played by OXA and OXB within the central nervous system.

Several other lines of evidence have also suggested that OXA and OXB may be differentially involved in particular functional systems. First, repeated studies have shown that OXA is more effective than OXB in promoting ingestive behavior [1,5,9]. This conclusion is supported by data from orexin receptor studies. Orexin A-induced ingestive behavior is attenuated by OX_1_R antagonists [15,36], and food deprivation selectively up-regulates OX_1_R mRNA in the amygdala without affecting OX_2_R mRNA in this structure [34]. Second, although OXB is generally ineffective in eliciting feeding or drinking behavior, there is evidence that OXB may be important in the promotion of arousal. Several studies have shown that the effects of orexins on arousal in thalamic midline and raphé nuclei depend primarily upon OX_2_R [37,38], and disruption of OX_2_R has been linked to the sleep disorder narcolepsy in dogs [39]. In at least one study, OXB has been shown to be more effective than OXA in activation of wakefulness-promoting thalamic nuclei in the rat [37], although this is not likely to be the case for all structures involved in the regulation of sleep and wakefulness, [33]. While both orexins are clearly involved in a multitude of homeostatic and regulatory processes, most published reports on their functions have not fully addressed potential differences between OXA and OXB.

Most published data on the distribution of orexin cells has focused on OXA, and relatively little is known about OXB. The distribution of OXB is generally described as being identical to that of OXA but is often presented incompletely [20,22,24] or not at all [19,21]. Because the two orexins are produced by the same precursor protein, there may have been a tendency in the early literature on the orexins to assume that the peptides are produced equally in each orexin cell, and at least one study has provided some support for this assumption [40]. However, there is evidence that the up-regulation of one orexin peptide over the other is very possible, and may serve some physiologically relevant function [23,41].

A second issue that has received little attention involves the tendency to assume that distributions of orexin-containing cells and fibers seen in one strain or species of research animal are representative of all strains and species. Although partial descriptions of orexin distribution within the central nervous system have been published for many species, including mice [42-44], cats [40,45], and humans [29], most studies examining orexin distribution in detail throughout the brain have been in rats (*Rattus norvegicus*). In addition, most descriptions of the orexins in the rat were performed using the highly inbred Wistar strain [19,20,24]. The occasional differences found in orexin distribution between rat strains tend to be largely ignored or overlooked. For example, OXA-IR cells have been described in the median eminence [21] in the Sprague-Dawley (SD) rat, a less inbred strain, but not in the Wistar rat. Although previous studies of the Wistar rat showed orexin-IR cell bodies only in the lateral hypothalamus, at least one study has shown OXB-IR, but not OXA-IR cells in the amygdala of both Wistar and SD rats [46]. These studies highlight both the importance of examining orexin distribution in more than one specific animal type, and the previously mentioned importance of examining both forms of orexin.

While the orexins have been studied in some depth in nocturnal laboratory rodents, little attention has been paid to them in diurnal animal models. Detailed descriptions of orexin cell or fiber distributions in diurnal animals are limited, and currently there are no descriptions of the distribution of both peptides in any diurnal species that are as complete as those available for the Wistar rat. Studies in humans [29] and sheep [47] describe OXA in limited portions of the brain, and the distribution of OXA-IR cell bodies has also been described in the Korean chipmunk (*Tamias sibiricus barberi*) [14] and the African green (vervet) monkey (*Cercopithecus aethiops*) [18]. The most complete study to date in a diurnal animal describes OXB, but not OXA in the Nile grass rat (*Arvicanthis niloticus*) [48]. Given the reported relationship between the orexins and regulation of sleep and arousal [reviewed in [49]], it is especially important to investigate potential differences in orexin distribution in animals with completely different sleep patterns, such as those seen in nocturnal and diurnal animals. This is especially important because the loss of orexin or the dysfunction of its receptors have been linked to several disorders in humans [50-53], a diurnal species.

One potential diurnal animal model with which to investigate this issue is the Nile grass rat, a rodent native to sub-Saharan Africa. The grass rat exhibits strong diurnal patterns of activity both in the laboratory and in the field [54,55]. The grass rat is a murid rodent, and as such is closely related to the standard laboratory rat (hereafter referred to as the lab rat). The grass rat is an excellent model for investigation of various physiological differences between nocturnal and diurnal animals, including those associated with sleep [56,57], reproduction [55,58-60], and the circadian regulation of activity [61-66]. We have recently demonstrated that the grass rat exhibits a diurnal rhythm in Fos-immunoreactivity in OXB cells [67].

A second diurnal animal model being used in laboratory study of circadian biology is the degu (*Octodon degus*), a highly social South American hystricomorph rodent [68]. The degu is relatively long-lived and matures slowly, unlike the rapidly maturing rats and mice commonly used in laboratory research [Reviewed in [69]]. Like hamsters, rhythms in degus are strongly influenced by social and olfactory cues [70,71], and there is some evidence for seasonal (photoperiodic) changes in reproductive structures [Reviewed in [69]]. The degu has been used as a model for research on sleep and the circadian regulation of behavior [72-78].

The location of orexin cell bodies may influence the constellation of signals reaching these cells, and the distribution of their fibers should reflect which brain regions receive arousal-inducing stimuli via the orexinergic system. The basic function of this system is likely to be very similar across species, as every animal must have the capacity to undergo arousal at particular times of day and in response to particular types of arousal-inducing stimuli. Indeed, published data show very similar distributions of orexin cells and fibers across species. However, animals are not the same with respect to all of the stimuli that produce arousal or to all of the responses associated with that arousal. There are therefore likely to be some differences among species with respect to which signals converge on orexin cells and to where these cells send their signals. To evaluate this hypothesis, we conducted a systematic analysis of the distribution and relative abundance of OXA and OXB cell bodies and fibers and systematically compared them in two diurnal species (the grass rat and the degu) and two nocturnal species, the lab rat and golden hamster (*Mesocricetus auratus*). As previous studies have already described orexin distributions in the highly inbred Wistar rat, we chose to use a less inbred strain, the Long-Evans (LE) rat, in the current study.

## Materials and methods

### Animal handling

Adult male grass rats (n = 4) and degus (n = 4) were obtained from captive breeding colonies at Michigan State University and the University of Michigan, respectively. Adult male Long Evans rats (n = 3) and hamsters (n = 4) were obtained from a commercial breeder (Charles River Laboratories, Raleigh, North Carolina). All animals were housed under standard light-dark (LD) cycles (LE rat, grass rat, and degu, 12:12 LD; hamster, 14:10 LD) with food and water provided *ad libitum *prior to perfusion. All animal handling procedures in this study followed National Institutes for Health guidelines and were approved by the Michigan State University All-University Committee for Animal Use and Care.

### Tissue collection and processing

All animals used in this study were sacrificed during the light phase of the LD cycle. At the time of sacrifice, all animals were anesthetized with sodium pentobarbital (Nembutal; Abbot Laboratories, North Chicago, IN) and perfused transcardially with 0.01 M phosphate-buffered saline (PBS; pH 7.4, 150–300 ml/animal), followed by 150 to 300 ml of fixative (4% paraformaldehyde in 0.1 M phosphate buffer, pH 7.4). Brains were post-fixed for 4 to 8 hours in 4% paraformaldehyde before being transferred to 20% sucrose in 0.1 M phosphate buffer. After 24 h in sucrose, brains were sectioned in three series at either 30 μm (grass rat and hamster) or 40 μm (LE rat and degu) using a freezing microtome.

Coronal sections from the olfactory bulb through the brain stem of four grass rats, three LE rats, four hamsters and four degus were used to determine orexin A and B fiber distribution. A third series of sections from one grass rat and one degu was stained with Cresyl violet, mounted on gelatin-coated slides and coverslipped to aid in delineation and identification of different structures. Additional sections through the preoptic area and lateral hypothalamus from two LE rats, three grass rats and one hamster were used in OXA blocking experiments. Tissue from the LH of all four species was used in OXA and OXB blocking experiments.

Tissue was processed for OXA or OXB immunoreactivity in the following manner. Unless otherwise specified, all steps were carried out at room temperature. Free-floating tissue was incubated in 5% normal donkey serum (NDS; Jackson Laboratories), in PBS with 0.3% Triton-X 100 (Research Products International, Mount Prospect, IL; PBS-TX) for 1 h. Tissue was then incubated in primary antibody for 42 h at 4°C (goat anti-orexin A 1:10,000, or goat anti-orexin B 1:10,000, Santa Cruz Biotechnology, Santa Cruz, CA; in PBS-TX and 3% NDS), and then in biotinylated secondary antibody for 1 h (donkey anti-goat 1:500, Santa Cruz; in PBS-TX and 3% NDS), followed by 1 h in avidin-biotin complex (0.9% each avidin and biotin solutions, Vector Laboratories, Burlingame, CA; in PBS-TX). Tissue was rinsed and reacted in diaminobenzidine (DAB, 0.5 mg/ml, Sigma) in a tris-hydrochloride buffer (Trizma, Sigma; pH 7.2) with hydrogen peroxide (0.35 μl 30% hydrogen peroxide/ml buffer). Control sections were incubated in the PBS-TX/NDS solution, with the primary antibody omitted. Tissue used in blocking experiments was processed as described above, but prior to adding tissue to the primary antibody solution, the primary antibody was preabsorbed with one or both orexin blocking peptides for 48 h at 4°C (1:50 OXA, 1:50 OXB, or 1:50 each OXA and OXB blocking peptide, Santa Cruz; in PBS with 0.3% Triton-X 100 and 3% NDS). All tissue was mounted on gelatin-coated slides, dehydrated, and coverslipped.

### Specificity of antibodies

The primary antibodies used in this study were obtained from Santa Cruz Biotechnology, Inc. (OXA, catalog number sc-8070; OXB, catalog number sc-8071). Each is a polyclonal, affinity-purified antibody raised against a peptide corresponding to a 19 amino acid sequence at the C-terminus of the respective orexin peptides. The blocking peptides used in this study, also obtained from Santa Cruz, are the peptide fragments against which the primary antibodies were raised. The specificity of these primary antibodies to their respective peptides has been established in previously published studies, using either preabsorption studies against the full-length orexin peptide [OXA; [79]]; or by comparison with antibodies of known specificity [OXB; [80]].

### Cell and fiber counts and analysis

For analysis of orexin A or B fiber distribution, all sections were examined under a light microscope (Leitz, Laborlux S, Wetzlar, Germany), and the presence or absence of labeled fibers, as well as their density, was recorded for brain regions from the olfactory bulbs through the brainstem. Tissue from a minimum of two animals were sampled for each region examined in this manuscript. The distribution of all OXA or OXB-IR cell bodies was also mapped for each species. High-resolution digital photographs of representative sections were taken using a digital camera (Carl Zeiss, AxioCam MRc; Göttingen, Germany) attached to a Zeiss light microscope (Axioskop 2 Plus). Image contrast and color balance were optimized using Zeiss AxioVision software (Carl Zeiss Vision). Final figures were prepared using Adobe Illustrator and Adobe Photoshop (Adobe Systems, San Jose, CA).

To systematically record the distribution and relative density of OXA and OXB fibers within each species, fiber densities were divided into the following five categories: very dense (++++), dense (+++), moderately dense (++), low density (+), and absent (-). To control for effects of potential species differences in the affinity of the antibodies for their antigens, or for differences associated with the thickness of the sections used for the different species, this study compares relative densities within each species rather than quantitative measurements across all species. In each species examined, "very dense" is defined as the region with the greatest density of orexin fibers in that species (typically the locus coeruleus), and "low density" defined as areas in which fibers are sparsely distributed (as in the cortex). Terminology and abbreviations for neural structures in this study follow Paxinos and Watson [81], except for divisions of the hamster bed nucleus of the stria terminalis (BNST) which follows Morin and Wood [82]. Comparisons of BNST divisions between species follow the comparison of terminologies between rat and hamster outlined in Alheid et al. [83]. Stereotaxic atlases for the rat [81] and Syrian hamster [82] were used to aid in identification of specific structures in these species. Because no available atlases for the degu and grass rat contain the level of detail necessary for this study, Nissl-stained tissue was used in concert with the rat atlas [81] for identification of nuclei in these species. Functional and anatomical grouping of brain structures for analysis followed Paxinos [84], as modified by John I. Johnson (personal communication). Because of the resulting size and complexity of the orexin fiber data set, fiber densities in the regions examined in this study were subjected to a principal components factor analysis (PCA; Statistica, StatSoft, Tulsa, OK) to identify patterns in the data.

## Results

### Orexin cell bodies

#### General

**Figure 1:**
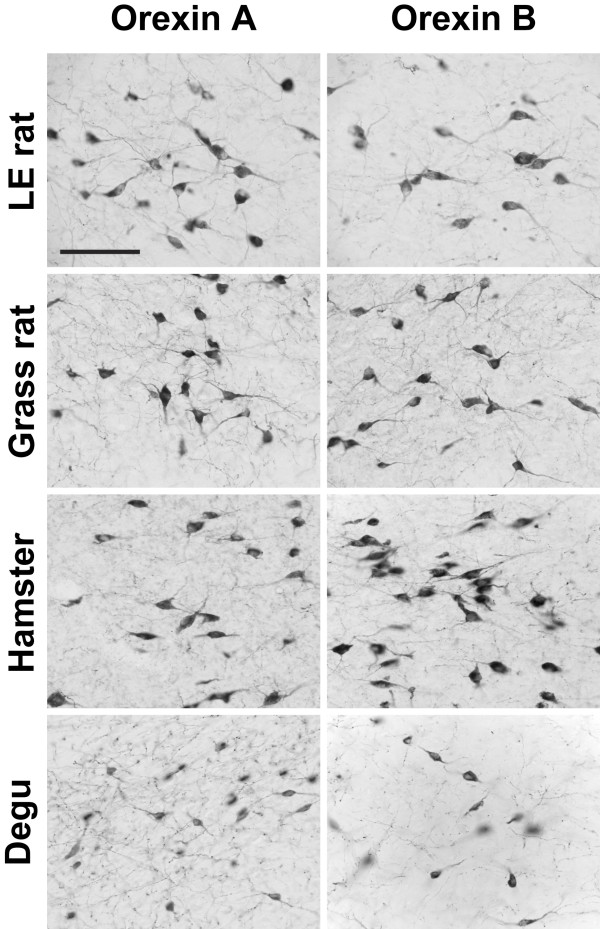
**Orexin A and B cell bodies**. Photomicrographs of orexin A and orexin B cell bodies in the Long-Evans rat, grass rat, Syrian hamster, and degu. Scale bar = 100 μm.

**Figure 2:**
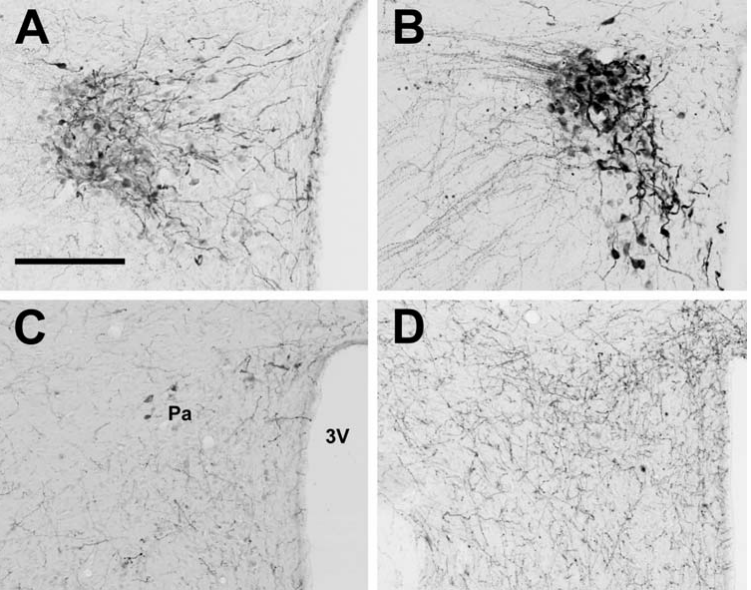
**Paraventricular nucleus**. Photomicrographs of orexin A cell bodies in the paraventricular nucleus (Pa) of the Long-Evans rat (**A**), grass rat (**B**), and Syrian hamster (**C**). Note lack of similar cell bodies in the degu (**D**). 3V: third ventricle. Scale bar = 200 μm.

**Figure 3:**
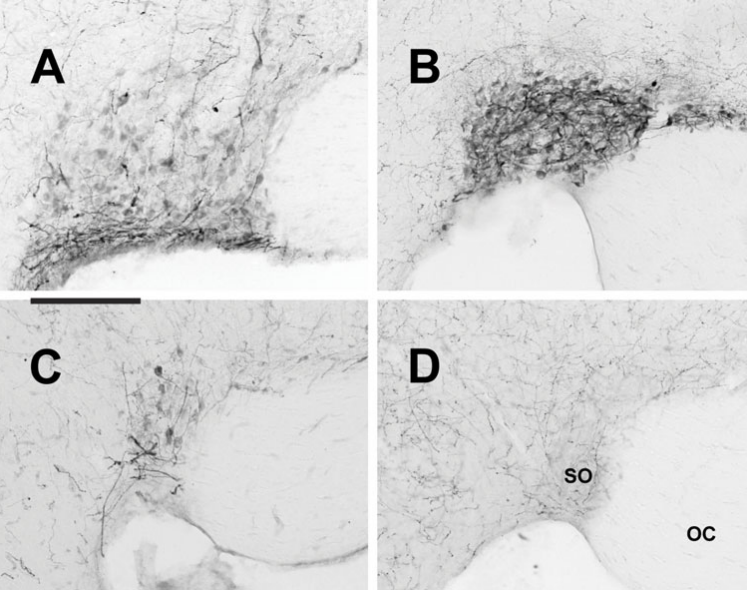
**Supraoptic nucleus**. Photomicrographs of orexin A cell bodies in the supraoptic nucleus (SO) of the Long-Evans rat (**A**), grass rat (**B**), and Syrian hamster (**C**). Note lack of similar cell bodies in the degu (**D**). OC: optic chiasm. Scale bar = 200 μm.

**Figure 4:**
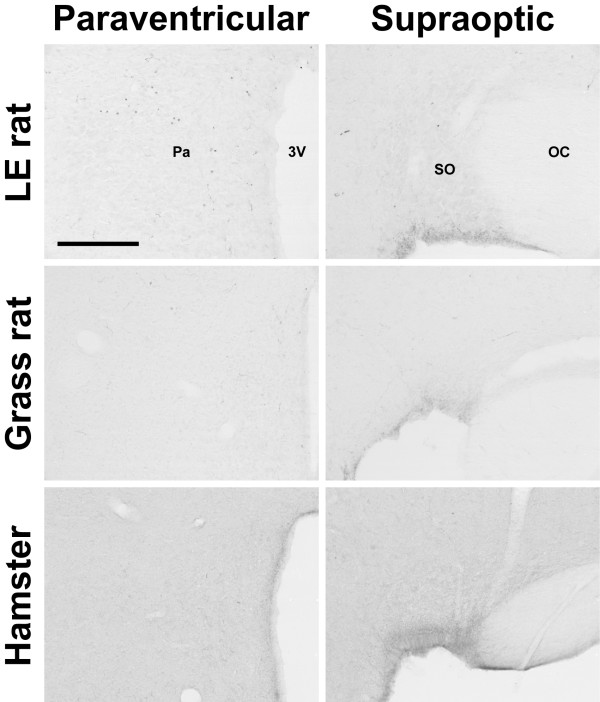
**Preabsorption of blocking peptide**. Photomicrographs of paraventricular nucleus (Pa; column 1) and supraoptic nucleus (SO; column 2) of the Long-Evans rat, grass rat, and Syrian hamster after preabsorption with orexin A blocking peptide. Note that preabsorption with blocking peptide eliminates cell bodies seen in Figures 2 and 3. 3V: third ventricle; OC: optic chiasm. Scale bar = 200 μm.

**Figure 5:**
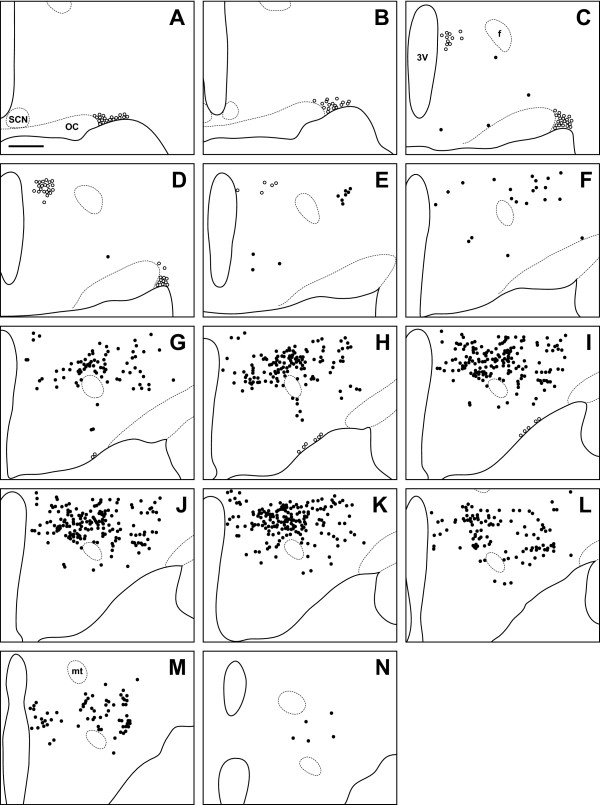
**Long-Evans rat**. Line drawing of every 6th section through the region of the Long-Evans rat hypothalamus that contains orexin cells. Sections are ordered from rostral (**A**) to caudal (**N**). Filled circles indicate locations where both orexin A and orexin B neurons are found, while open circles indicate orexin A neurons only. 3V: third ventricle; SCN: suprachiasmatic nucleus; OC: optic chiasm; f: fornix; mt: mammillothalamic tract. Scale bar = 500 μm.

**Figure 6:**
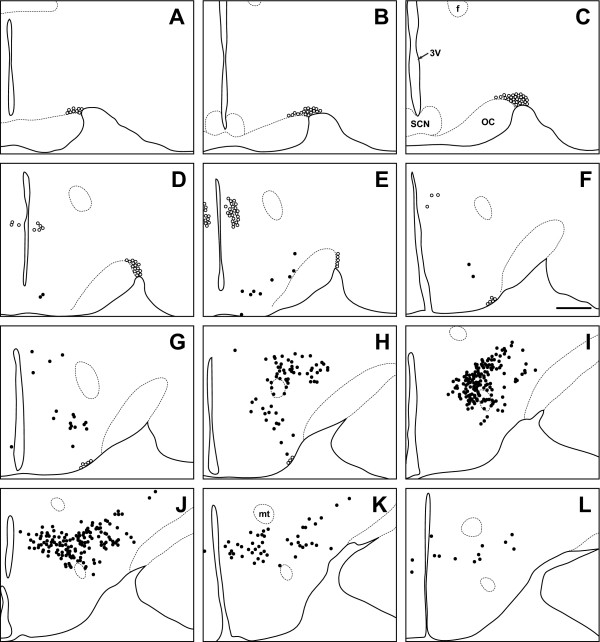
**Grass rat**. Line drawing of every 6th section through the region of the grass rat hypothalamus that contains orexin cells. Sections are ordered from rostral (**A**) to caudal (**L**). Filled circles indicate locations where both orexin A and orexin B neurons are found, while open circles indicate orexin A neurons only. 3V: third ventricle; SCN: suprachiasmatic nucleus; OC: optic chiasm; f: fornix; mt: mammillothalamic tract. Scale bar = 500 μm.

**Figure 7:**
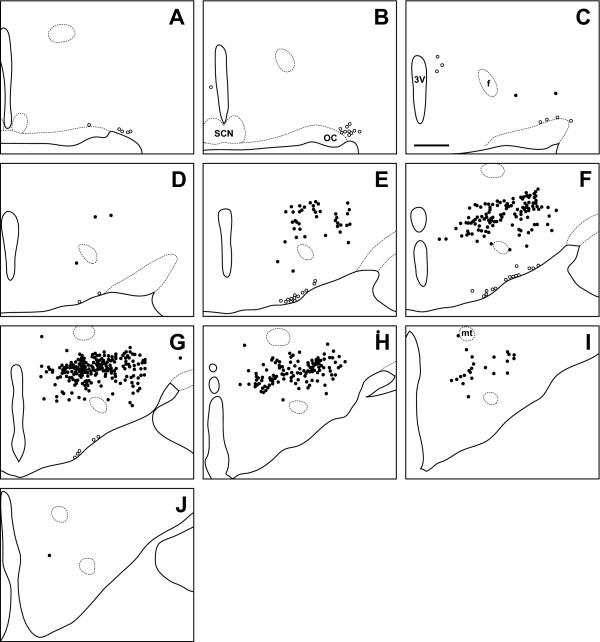
**Syrian hamster**. Line drawing of every 6th section through the region of the Syrian hamster hypothalamus that contains orexin cells. Sections are ordered from rostral (**A**) to caudal (**J**). Filled circles indicate locations where both orexin A and orexin B neurons are found, while open circles indicate orexin A neurons only. 3V: third ventricle; SCN: suprachiasmatic nucleus; OC: optic chiasm; f: fornix; mt: mammillothalamic tract. Scale bar = 500 μm.

**Figure 8:**
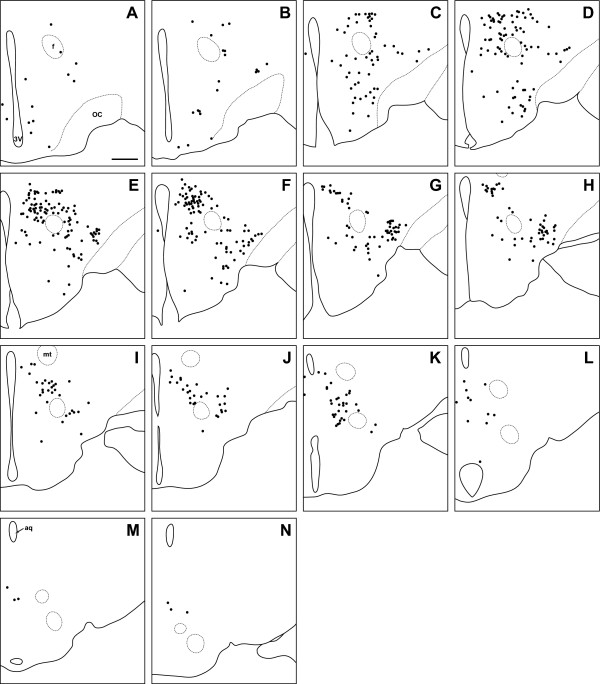
**Degu**. Line drawing of every 6th section through the region of the degu hypothalamus that contains orexin cells. Sections are ordered from rostral (**A**) to caudal (**N**). Filled circles indicate locations where both orexin A and orexin B neurons are found. 3V: third ventricle; OC: optic chiasm; f: fornix; mt: mammillothalamic tract; aq: cerebral aqueduct. Scale bar = 500 μm.

In all animals examined, OXA- and OXB-IR cell bodies were present in the lateral hypothalamus (Figure 1). Within each species examined, the distribution of orexin-IR neurons was quite consistent, with little individual variation from one animal to the next. Although the distribution of orexin neurons differed among species, the majority of the orexin cells in all species were observed in the perifornical region (PeF) and lateral hypothalamic area (LHA). In the LE rat, grass rat and hamster, cell bodies immunoreactive for OXA were also found in the paraventricular hypothalamic nucleus (Pa), supraoptic nucleus (SO), and the supraoptic retrochiasmatic nucleus (SOR) (Figure 2, Figure 3). Orexin A-IR neurons in the Pa and SO were generally smaller and less intensely stained for OXA than were cells in the PeF. For all three species exhibiting orexin-IR neurons in the Pa and SO, the OXA-IR nuclei did not appear to represent nonspecific binding of the primary antibody, as preabsorption of the primary antibody with OXA blocking peptide supplied by the manufacturer reduced or eliminated OXA immunoreactivity in these nuclei (Figure 4). Preabsorption with blocking peptides reduced or eliminated both OXA and OXB immunoreactivity in the LH in all four species examined (not shown). No OXB-IR neurons were observed in Pa, SO, or SOR of any species. Orexin A and OXB cell body distribution in the LE rat, grass rat, degu and hamster are summarized in Table 1 and are depicted in Figures 5, 6, 7, and 8, respectively.

**Table 1:** Orexin cell body distribution

**Brain region**	**LE rat**		**Grass rat**		**Hamster**		**Degu**	
	**A**	**B**	**A**	**B**	**A**	**B**	**A**	**B**
Dorsal hypothalamic area	+	+	+	+			++	++
Dorsomedial hypothalamic nucleus	+++	++	++	++	++	++	++++	++++
Lateral hypothalamic area	+++	+++	+++	+++	++++	++++	++++	++++
Paraventricular hypothalamic nucleus, magnocellular	+++	-	++++	-	-	-	-	-
Perifornical nucleus	++++	++++	++++	++++	+	+	++	++
Posterior hypothalamic area	++	++	+++	+++	-	-	++	++
Retrochiasmatic area	+	+	+	+	-	-	+	+
Subincertal thalamic nucleus	+	+	++	+	+	+	+	+
Supramammillary nucleus		-		-	-	-	+	+
Supraoptic nucleus	++++	-	++++	-	++	-	-	-
Supraoptic nucleus, retrochiasmatic	++	-	+++	-	+	-	-	-
Tuberum cinerum	++	++	+++	+++	+	+	++	++

Distribution of orexin A and B cell bodies in the Long-Evans rat, grass rat, Syrian hamster, and degu. The relative densities of OXA- or OXB-containing cells are indicated in each column as very dense (++++), dense (+++), moderately dense (++), sparse (+), or absent (-).

### Orexin cell bodies in the LE Rat

In the LE rat, OXA- and OXB-IR cells were observed at high density in the PeF, LHA, and dorsomedial hypothalamic nucleus (DMH) (Figure 5). Moderate densities of labeled cells were present in the posterior hypothalamic area (PH) and tuberum cinerum (TC). Sparsely scattered cells were also observed in the dorsal hypothalamic area (DH), retrochiasmatic area (RCh), and the subincertal thalamic nucleus (SubI). Orexin A-IR cell bodies in SO and magnocellular Pa were very dense (Figure 2A, Figure 3A), while cell bodies in SOR were only moderately dense No OXB-IR neurons were seen in the Pa or SO.

### Orexin cell bodies in the grass rat

The distribution of OXA- and OXB-IR neurons in the grass rat was similar to the pattern seen in the LE rat. Cell bodies expressing OXA and OXB were observed at very high density in PeF, TC, LHA, and PH (Figure 6). Unlike the LE rat, small numbers of OXA- and OXB-IR neurons in the PeF were observed inside the fornix in all grass rats examined in this study. Moderate densities of OXA- and OXB-IR cell bodies were visible in DMH and SubI, with sparsely scattered cells also present in RCh and DA. As in the LE rat, cell bodies expressing strong OXA immunoreactivity were present in PA, SO, and SOR (Figure 2B, Figure 3B). No OXB-IR neurons were present in these nuclei. Orexin A-IR cell bodies were very densely distributed in the magnocellular Pa and SO. Moderately dense OXA-IR cell bodies were present in SOR.

### Orexin cell bodies in the hamster

Unlike the LE rat and grass rat, OXA- and OXB-IR neurons in the hamster were conspicuously absent in PeF; the majority of orexin cells were observed in LHA and DMH, dorsal or dorsomedial to PeF, with sparsely scattered cells also present in TC and SubI (Figure 7). Although OXA-IR neurons were present in the hamster magnocellular PA, SO, and SOR (Figure 2C, Figure 3C), the overall density of OXA-IR cell bodies in these structures was far lower than that in the LE rat or grass rat. As in the LE rat and grass rat, no OXB-IR neurons were present in these nuclei.

### Orexin cell bodies in the degu

The distribution of OXA- and OXB-IR cell bodies was very different in the degu than in the LE rat, grass rat and hamster. The regions of highest density of OXA and OXB cells formed two distinct clusters, in the DMH and in the LHA ventrolateral to the fornix (Figure 8). Orexin A and OXB neurons were less densely distributed in the PeF, DA, and PH. In the PeF, orexin-IR neurons were clustered tightly against the boundaries of the fornix, but did not intrude into this structure as observed in the grass rat. Cells labeled for OXA and OXB were sparsely distributed throughout the RCh and SubI. Unlike the hamster, grass rat and LE rat, no orexin-IR neurons were visible in the Pa, SO, or SOR (Figure 2D, Figure 3D). Orexin A- and B-IR cells in the degu were also different from those seen in the other three species in that orexin neurons were scattered through the TC caudal to the ventromedial hypothalamic nucleus (VMH), with small numbers of cells observed near the midline in sections through the supramammillary nucleus (SuM).

### Orexin fiber distribution

#### General

Orexin A- and OXB-IR fibers were visible in many regions throughout the forebrain, midbrain, and hindbrain in all species examined (Table 2). Numerous varicosities were noted on these fibers, especially in the most dense projections extending rostrally from the lateral hypothalamus to the preoptic area and amygdala, and posteriorly through the periaqueductal gray to the raphé nuclei and brainstem. The average density of OXA and OXB fiber innervation in over two hundred individual brain regions was examined for each species. A PCA analysis of these fiber densities revealed only one significant eigenvector (eigenvalue = 5.740, cumulative variance explained = 71.748%); no other factors with eigenvalues greater than 1 were revealed. Factor coordinates for all species along this eigenvector were similar (LE Rat OXA = -0.8753, OXB = -0.8669; Grass rat OXA = -0.8275, OXB = -0.8178; Hamster OXA = -0.8657, OXB = -0.8446; Degu OXA = -0.8270, OXB = -0.8498), suggesting a strong overall similarity between OXA and OXB fiber densities across species. Overall, the PCA analysis revealed that correlations in OXA measurements between pairs of species ranged from 59.2% to 72.2% similarity, while these measurements for OXB ranged from 57.1% to 68.6%. The PCA analysis thus suggests that the four species were very similar with respect to their relative distributions of orexinergic fibers across the brain, but that there are also some very real differences. Within species, correlations between OXA and OXB fiber densities were extremely high (LE rat: 85.8%; grass rat: 85.2%; hamster: 92.4%; degu: 98.5%). Significance levels for all correlations were < 0.001. The PCA correlation matrix summarizing between-species correlations for OXA and OXB is presented in Table 3. The overall details of the distribution and relative density of OXA and OXB fibers for each species are summarized in Table 2. Below we provide a general description of the patterns of fiber distribution and highlight the differences between species.

**Table 2:** Orexin fiber distribution

**Brain region**	**LE rat**		**Grass rat**		**Hamster**		**Degu**	
	**A**	**B**	**A**	**B**	**A**	**B**	**A**	**B**
**Hypothalamus**								
Anterior hypothalamic area	++	++	+	+	+++	++	+++	+++
Anterodorsal preoptic nucleus	+	+	+	+	++	+	++	++
Anteroventral periventricular nucleus	++	+	+	-	++	++	++	++
Arcuate nucleus	+++	+++	+++	+	++	++	++	++
Circular nucleus	++	+	++	+	+++	+++	+++	+++
Dorsal hypothalamic area	++	++	++	++	++	++	++	++
Dorsomedial nucleus	+++	++	++	+	+++	++	+++	+++
Lateral hypothalamic area	+++	+++	+++	+++	+++	+++	++	++
Lateral mammillary nucleus	+	+	+	+	+++	+++	+	+
Lateral preoptic area	+++	+	+	+	++	+	++	++
Lateroanterior nucleus	++	+	++	++	+++	+++	+++	+++
Magnocellular preoptic nucleus	+	+	+	+	++	++	++	++
Medial mammillary nucleus, lateral part	-	-	-	-	-	-	+	+
Medial mammillary nucleus, medial part	-	-	-	-	-	-	-	-
Medial mammillary nucleus, median part	+	+	+	+	-	-	+	+
Medial preoptic area	++	+	+	+	++	++	++	++
Medial preoptic nucleus	++	+	++	++	++	+	++	++
Median preoptic nucleus	+++	+++	++	++	+++	+++	+++	+++
Parastrial nucleus	+++	++	++	++	+	+	+	+
Paraventricular nucleus, magnocellular	++++	++	++	+	++	++	++	++
Paraventricular nucleus, parvocellular	+++	++	+++	++	++	++	++	++
Perifornical nucleus	++++	+++	+++	++	+	+	+++	+++
Periventricular nucleus	+++	++	++	+	++	++	+++	+++
Posterior hypothalamic area	+++	+++	++	++	++	++	++	++
Posterodorsal preoptic nucleus	++	+	++	++	++	+	++	++
Preoptic nucleus, ventromedial	+++	+++	+++	++	++	++	+++	+++
Retrochiasmatic area	+++	++	++	++	+++	++	+++	+++
Subincertal nucleus	+++	+++	++	++	+	+	+	+
Submammillothalamic nucleus	++	+	++	++	+	+	++	++
Suprachiasmatic nucleus, anterior	-	-	-	-	+	+	-	-
Suprachiasmatic nucleus, posterior	+	+	-	-	+	+	+	+
Supramammillary nucleus	+++	++	++	+	++	++	+++	+++
Supraoptic nucleus	++++	+	++++	++	++	+	+	+
Supraoptic nucleus, retrochiasmatic	++++	++	+++	+	++	+	+++	+++
Tuberomammillary nucleus	++++	++++	+++	+++	++++	++++	++++	++++
Tuberum cinereum	++	++	+++	++	+	+	++	++
Ventrolateral preoptic area	+++	++	+	+	+++	++	+++	+++
Ventromedial nucleus	++	+	+	+	++	++	++++	++++
Ventromedial nucleus, anterior	+	+	+	+	++	+	++++	++++
Zona incerta, caudal	++	+	+	+	+	+	-	-
Zona incerta, rostral	++	+	+	+	++	++	+	+
**Thalamus**								
Anterodorsal nucleus	+	+	++	++	+	+	+	+
Anteromedial nucleus	-	-	-	-	-	-	-	-
Anteroventral nucleus	+	+	+	-	-	-	-	-
Central medial nucleus	++	++	+	+	+	-	++	+
Centrolateral nucleus	+	+	+	+	+	+	+++	+++
Dorsolateral geniculate nucleus	-	-	-	-	-	-	-	-
Interanterodorsal nucleus	+	+	+	+	++	+	-	-
Interanteromedial nucleus	++	+	+	+	+	+	-	-
Intergeniculate leaflet	++	++	++	++	++	++	++	++
Intermediodorsal nucleus	++	++	+++	+++	++	++	+	+
Lateral posterior nucleus	-	-	-	-	-	-	-	-
Laterodorsal thalamic nucleus	+	+	-	-	-	-	-	-
Medial geniculate nucleus	-	-	+	+	-	-	-	-
Mediodorsal thalamic nucleus	+	+	+	+	++	+	++	++
Nucleus of the fields of Forel	+	+	-	-	+	+	-	-
Paracentral nucleus	++	++	-	-	+	+	+++	+++
Parafascicular nucleus	+	+	+	+	+	-	-	-
Paratenial nucleus	-	-	++	+	+	+	+	+
Paraventricular nucleus	++++	++++	+++	+++	++++	++++	++++	++++
Posterior intralaminar nucleus	++	++	+	+	+	+	+	+
Posterior limitans	++	++	++	+	++	++	++	++
Posterior nuclear complex	-	-	-	-	-	-	-	-
Reticular thalamic nucleus	+	-	-	-	-	-	-	-
Reuniens nucleus	+	+	+	+	+	-	+	+
Rhomboid nucleus	++	++	-	-	++	++	-	-
Submedius nucleus	-	-	+	+	+	-	-	-
Subparafascicular nucleus	+	+	+	+	+	+	+	+
Ventral anterior nucleus	-	-	-	-	-	-	-	-
Ventral posteriolateral nucleus	-	-	-	-	-	-	-	-
Ventral posteriomedial nucleus	-	-	-	-	-	-	-	-
Ventrolateral geniculate nucleus	+	+	+	+	+	+	+	+
Ventrolateral thalamic nucleus	-	-	-	-	-	-	-	-
Ventromedial thalamic nucleus	++	+	-	-	-	-	-	-
Xiphoid nucleus	+++	++	+++	++	+++	+++	+	+
**Epithalamus**								
Lateral habenular nucleus	++	+	-	-	+	+	+	+
Medial habenular nucleus	-	-	++	+	++	-	-	-
Stria medullaris	-	-	-	-	+	-	-	-
**Cerebral isocortex**								
Layer 1	+	+	+	+	+	+	+	+
Layer 2	+	+	+	+	+	+	+	+
Layer 3	-/+	-/+	-/+	-/+	-/+	-/+	-/+	-/+
Layer 4	-/+	-/+	-/+	-/+	-/+	-/+	-/+	-/+
Layer 5	+	+	+	+	+	+	+	+
Layer 6	+	+	+	+	+	+	+	+
Agranular insular cortex	++	+	+	+	+	+	+	+
Cingulate and retrosplenial agranular cortex	+	+	+	+	+	+	-	-
Frontal cortex	+	+	+	+	+	+	+	+
Granular insular cortex	+	+	+	+	++	++	+	+
Occipital cortex	-	-	+	+	+	+	+	+
Orbital cortex	+	+	+	+	+	+	+	+
Parietal cortex	+	+	+	+	+	+	+	+
**Amygdala**								
Anterior amygdaloid area	+	+	+	+	++	++	++	++
Anterior, posteromedial, and posterolateral cortical amygdaloid nuclei	++	++	++	++	+	+	++	++
Basolateral amygdaloid nucleus	+	+	-	-	+	+	+	+
Basomedial amygdaloid nucleus	+	+	+	+	+	+	++	++
Bed nucleus of the stria terminalis*								
Intraamygdaloid division	+	+	+	+	+	+	+	+
Lateral division	++	++	++	+	N/A	N/A	+	+
Lateral division, dorsal (Rat), anterolateral (Hamster)	++	+	+	+	++	++	+	+
Lateral division, posterior (Rat), posterolateral (Hamster)	++	++	++	+	++	++	+	+
Lateral division, ventral (Rat), anteroventral (Hamster)	+++	+++	++	+	++	++	++	++
Medial division, anterior (Rat), anteromedial (Hamster)	++	++	++	++	++	++	++	++
Medial division, posterointermediate (Rat), posteromedial (Hamster)	+	+	++	+	++	++	++	++
Medial division, posterolateral (Rat), posterointermediate (Hamster)	+	+	++	+	++	++	++	++
Central amygdaloid nucleus	+	+	+	+	+	+	-	-
Intercalated amygdaloid nucleus	++	+	+	-	++	++	++	++
Lateral amygdalohippocampal area	+	+	+	-	+	+	++	++
Lateral amygdaloid nucleus	+	+	+	+	+	+	+	+
Medial amygdaloid nucleus	++	+	+	+	+++	+++	+	+
Nucleus of the lateral olfactory tract	+	+	+	+	++	++	+	+
Posteromedial amygdalohippocampal area	+	+	+	+	+	+	+++	+++
**Hippocampus**								
CA1	+	+	+	+	-	-	-	-
CA2	-	-	-	-	+	+	-	-
CA3	-	-	-	-	+	+	+	+
Dentate gyrus	+	+	-	-	-	-	+	+
Tenia tecta	+	+	++	+	++	++	++	++
Entorhinal cortex	+	+	+	-	+	+	+	+
Indusium griseum	++	++	+	+	++	++	-	-
Subiculum	+	+	-	-	-	-	+	+
**Olfactory**								
Anterior olfactory nuclei	++	++	++	++	++	++	+	+
Islands of Calleja	+	+	+	+	++	++	++	++
Olfactory bulb	-	-	-	-	-	-	-	-
Olfactory tubercule	-	-	-	-	+	+	+	+
Piriform cortex	+	+	+	+	+	+	+	+
**Other Forebrain**								
Bed nucleus of the anterior commissure	+++	++	++	++	++	++	++	++
Claustrum	++	++	+	+	++	++	+	+
Dorsal endopiriform nucleus	++	+	+	+	+	+	+	+
**Septal nuclei**								
Lateral septal nucleus, dorsal part	++	++	++	+	++	+	+	+
Lateral septal nucleus, intermediate part	+	+	+	-	+	+	-	-
Lateral septal nucleus, ventral part	+	+	++	+	++	++	+	+
Medial septal nucleus	++	++	+	+	+++	+++	++	++
Nucleus of the horizontal limb of the diagonal band of Broca	+++	++	+	+	++	++	++	++
Nucleus of the vertical limb of the diagonal band of Broca	+++	+	+	+	+++	++	++	++
Septofimbrial nucleus	+	+	+	+	++	++	+	+
Septohippocampal nucleus	++	+	+	+	+++	+++	++	++
Triangular septal nucleus	+	+	-	-	+	+	-	-
**Basal ganglia**								
Accumbens nucleus, core	-	-	-	-	+	+	-	-
Accumbens nucleus, shell	+	+	++	+	-	-	+	+
Accumbens shell, lateral	+	+	+	+	+	+	-	-
Caudate putamen	-	-	-	-	+	+	-	-
Dorsal peduncular pontine nucleus	++	+	++	++	+	+	+	+
Globus pallidus, lateral	+	+	+	+	-	-	-	-
Globus pallidus, medial	-	-	-	-	-	-	-	-
Pedunculopontine tegmental nucleus	++	++	++	+	++	++	++	++
Peripeduncular nucleus	+	+	+	+	++	++	+	+
Substantia innominata	++	++	+	+	++	++	++	++
Substantia nigra, compact part	+	+	+	+	+	+	+	+
Substantia nigra, lateral part	++	++	+	+	++	++	+	+
Substantia nigra, reticular part	-	-	-	-	-	-	-	-
Subthalamic nucleus	+	+	+	+	++	++	-	-
Ventral pallidum	++	++	+	+	+	+	+	+
Ventral tegmental area	++	+	++	+	++	++	++	++
Ventral tegmental nucleus	++	+	-	-	+	+	+	+
**Brainstem reticular nuclei**								
B9 serotoin cells	++	++	+	+	++	++	-	-
Cuneiform nucleus	++	++	++	+	++	++	+	+
Deep mesencephalic nucleus	++	++	+	+	++	+	+	+
Dorsal paragigantocellular nucleus	+	+	-	-	+	+	+	+
Gigantocellular reticular nucleus	++	+	+	+	+	+	+	+
Gigantocellular reticular nucleus, ventral part	++	+	++	+	++	++	++	++
Gigantocellular reticular nucleus, α part	++	++	++	+	++	++	++	++
Intermediate reticular nucleus	+++	+	-	-	++	+	+	+
Lateral paragigantocellular nucleus	+++	++	+	+	++	++	+++	+++
Lateral reticular nucleus	++	+	+	+	+	+	+	+
Medullary reticular nuclei, dorsal part	+	+	+	+	+	+	+	+
Medullary reticular nuclei, ventral part	+	+	+	+	+	+	+	+
Paramedian reticular nucleus	+	+	-	-	+	+	+	+
Parvocellular reticular nucleus	++	++	+	+	++	+	+	+
Pontine reticular nucleus, caudal part	+	+	+	-	+	+	+	+
Pontine reticular nucleus, oral part	++	+	+	+	+	+	-	-
Pontine reticular nucleus, ventral part	-	-	+	+	++	++	+	+
Reticulotegmental nucleus of the pons	+++	++	++	++	+	+	+	+
Subcoeruleus nucleus, dorsal part	++	++	+	+	++	++	++	++
Subcoeruleus nucleus, ventral part	++	++	+	-	++	++	+	+
Subcoeruleus nucleus, á part	+++	+++	+++	++	+++	+++	+++	+++
**Sensory, somatic and motor nuclei**								
Abducens nucleus (VI)	-	-	-	-	-	-	-	-
Ambiguus nucleus	++++	+++	+++	++	++	+	++	++
Barrington's nucleus	++++	+++	+++	+++	++++	++++	+++	+++
Dorsal motor nucleus of vagus (X)	+	+	+	+	++	+	+	+
Facial nucleus (VII)	++	+	++	+	+	+	++	++
Gracile nucleus	-	-	-	-	++	+	+	+
Hypoglossal nucleus (XII)	+	+	+	-	-	-	+	+
Kölliker-Fuse nucleus	+++	++	++	++	+++	+++	+++	+++
Lateral parabrachial nucleus	+++	+++	++	++	++	++	++	++
Medial parabrachial nucleus	+++	++	+	+	++	+	++	++
**Nucleus of the solitary tract**								
Central	++	++	+	+	+	+	++	++
Dorsal	++++	+++	++++	++++	++++	++++	+++	+++
Medial	+	+	+++	++	+++	+++	+++	+++
Ventrolateral	+++	++	+	+	++	++	++	++
Oculomotor nucleus (III)	-	-	-	-	-	-	-	-
Trigeminal motor nucleus (V)	+	+	+	+	-	-	+	+
Trigeminal principal sensory nucleus (V)	+	+	+	+	+	+	+	+
Trigeminal spinal nucleus, caudal part	+	+	++	++	+	+	++	++
Trigeminal spinal nucleus, interpolar part	-	-	+	+	+	+	+	+
Trigeminal spinal nucleus, oral part	-	-	+	+	+	+	++	++
Trochlear motor nucleus (IV)	+	+	-	-	+	+	-	-
**Raphé nuclei**								
Dorsal raphé nucleus	++++	++++	+++	+++	++++	++++	++++	++++
Linear nucleus of the raphé	+++	+++	++	+	+++	++	++	++
Median raphé nucleus	++++	++	+++	+++	+++	+++	+++	+++
Pontine raphé nucleus	+++	+++	++	++	+++	++	+++	+++
Raphé magnus nucleus	+++	++	++	++	++	++	++	++
Raphé obscurus nucleus	+++	++	++	+	+++	+++	+++	+++
Raphé pallidus nucleus	+++	++	++	+	++	++	+	+
Rhabdoid nucleus	+	+	+	+	+	+	+	+
**Periaqueductal gray**								
Dorsolateral periaqueductal gray	++	++	+	+	++	++	+	+
Dorsomedial periaqueductal gray	+++	+++	++	++	+++	+++	++	++
Lateral periaqueductal gray	+++	+++	++	++	++	++	++	++
Thalamic periaqueductal gray	++	++	+	+	+++	++	++	++
Ventrolateral periaqueductal gray	+++	++	+++	++	+++	++	+++	+++
**Other midbrain**								
Dorsolateral pontine nucleus	+	+	++	++	-	-	+	+
Inferior colliculus	+	+	-	-	++	+	++	++
Interpeduncular nucleus	+	+	++	+	++	++	-	-
**Pretectal nuclei**								
Anterior pretectal nucleus	+	+	+	+	-	-	+	+
Medial pretectal nucleus	++	++	++	+	++	++	++	++
Posterior pretectal nucleus	++	+	+	+	-	-	++	++
Red nucleus	-	-	-	-	-	-	+	-
Superior colliculus	+	+	-	-	-	-	+	+
**Tegmental nuclei**								
Anterior tegmental nucleus	+	+	-	-	+	+	+	+
Dorsal tegmental nucleus	-	-	-	-	-	-	-	-
Laterodorsal tegmental nucleus	++	++	++	++	++	++	+++	+++
**Cerebellum**								
Cerebellar cortex	-	-	-	-	-	-	++	+
Interposed, lateral, and medial cerebellar nuclei	+	+	+	+	-	-	++	++
**Other hindbrain**								
Central tegmental tract	++	++	+	+	++	+	+	+
**Cochlear nuclei**								
Dorsal cochlear nucleus	+	+	-	-	+	+	+	+
Ventral cochlear nucleus	-	-	-	-	-	-	-	-
Inferior olive	++	+	+++	++	-	-	-	-
Lateral lemniscus	+	+	-	-	+	-	-	-
Locus coeruleus	++++	++++	++++	++++	++++	++++	+++	+++
Nucleus of Darkschewitsch	-	-	++	+	-	-	+	+
Parabigeminal nucleus	+	+	+	+	++	++	+++	++
Prepositus nucleus	++	+	-	-	+	+	++	++
**Superior olivary nuclei**								
Nucleus of the trapezoid body	++	+	+	+	-	-	+	+
Superior olive	-	-	-	-	-	-	-	-
Trapezoid body	-	-	+	+	+	+	-	-
Vestibular nuclei	+	+	+	+	+	-	++	+
**Circumventricular and non-neuronal**								
Area postrema	-	-	-	-	-	-	++	++
Choroid plexus	+	+	+	+	+	+	+	+
Ependyma	+	+	++	+	+	+	+	+
Median eminence	++++	+	++++	+	++	++	++	++
Subcommisural organ	-	-	-	-	-	-	-	-
Subfornical organ	+	+	+	-	-	-	++	+

Distribution of orexin A and B fibers in the Long-Evans rat, grass rat, Syrian hamster, and degu. The relative densities of OXA- or OXB-containing fibers are indicated in each column as very dense (indicated by ++++), dense (+++), moderately dense (++), sparse (+), or absent (-). Unmarked rows indicate areas for which no data are available.

**Table 3:** Principal component factor analysis

	**Orexin A**			
	**LE rat**	**Grass rat**	**Hamster**	**Degu**
**LE rat**	1.0000			
**Grass rat**	0.7220	1.0000		
**Hamster**	0.6982	0.6086	1.0000	
**Degu**	0.6412	0.5706	0.6458	1.0000

	**Orexin B**			
	**LE rat**	**Grass rat**	**Hamster**	**Degu**

**LE rat**	1.0000			
**Grass rat**	0.6989	1.0000		
**Hamster**	0.6862	0.6054	1.0000	
**Degu**	0.6321	0.5709	0.6401	1.0000

Pairwise correlation matrix of orexin fiber distribution in the Long-Evans rat, grass rat, Syrian hamster, and degu. Correlation values were obtained from a principal component factor analysis of orexin A and orexin B fiber density patterns in all four species. Values listed at intersections between rows and columns represent the correlation in fiber density between species indicated by the corresponding row and column labels. All values represent significant correlations (p < 0.001).

#### Hypothalamus

**Figure 9:**
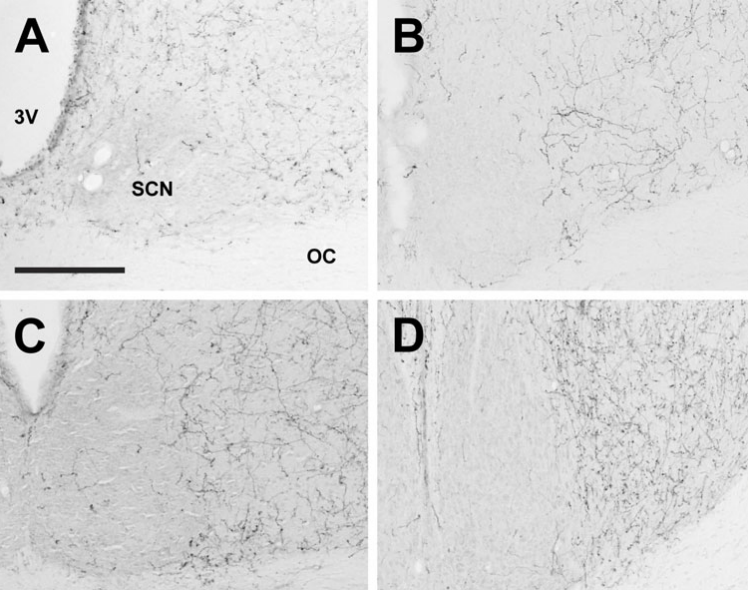
**Suprachiasmatic nucleus**. Photomicrographs of orexin A fibers around the suprachiasmatic nucleus (SCN) of the Long-Evans rat (A), grass rat (B), Syrian hamster (C), and degu (D). 3V: third ventricle; OC: optic chiasm. Scale bar = 200 μm.

**Figure 10:**
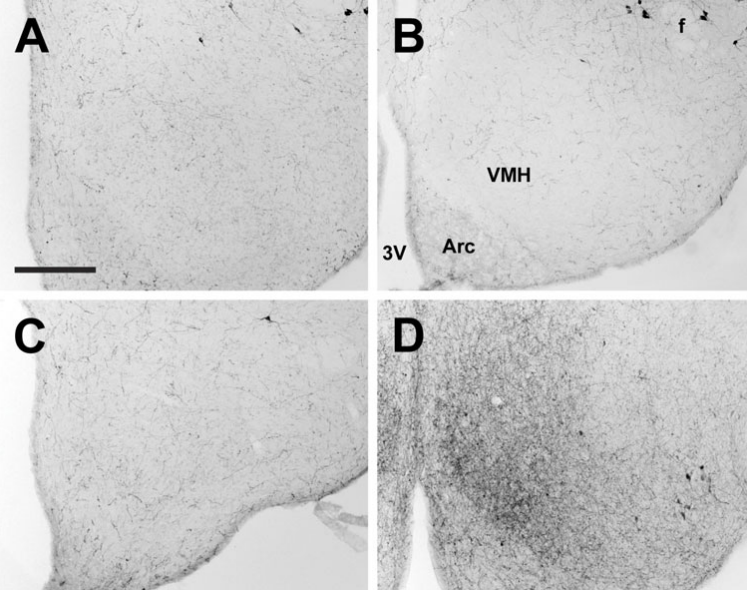
**Ventromedial hypothalamic nucleus**. Photomicrographs of orexin A fibers in the ventromedial hypothalamic nucleus (VMH) of the Long-Evans rat (A), grass rat (B), Syrian hamster (C), and degu (D). Note markedly higher density of orexin fibers in the degu VMH in comparison with the other three species. 3V: third ventricle; Arc: arcuate nucleus. Scale bar = 300 μm.

**Figure 11:**
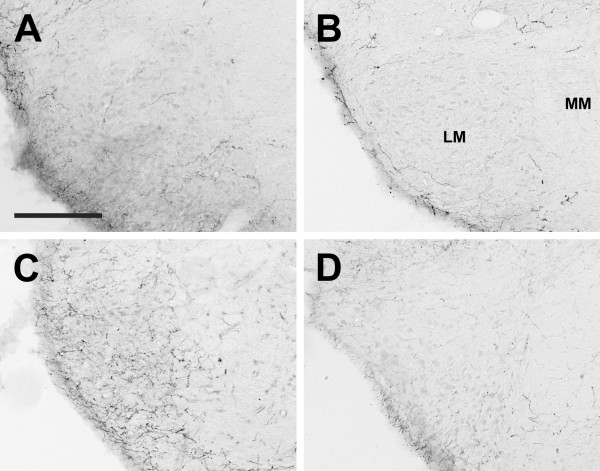
**Lateral mammillary nucleus**. Photomicrographs of orexin A fibers in the lateral mammillary nucleus (LM) of the Long-Evans rat (A), grass rat (B), Syrian hamster (C), and degu (D). Note higher density of orexin fibers in the hamster LM in comparison with the other three species. MM: medial mammillary nucleus. Scale bar = 200 μm.

In all species examined, OXA- and OXB-IR fibers extended from the lateral hypothalamus throughout much of the brain. These fibers were moderately to densely distributed throughout much of the hypothalamus, with the exception of the zona incerta, where fiber density was low, and the suprachiasmatic (Figure 9) and medial mammilary nuclei, which exhibited little to no orexin fibers in any species. Several species differences were observed. First, differences in fiber density were seen in the main regions in which we saw substantial differences in the distribution of labeled cells. In the hamster PeF, fiber density was reduced in comparison with the other species, and in the LE rat and grass rat, many OXA fibers were also present in the magnocellular Pa and SO. Orexin B fibers in all species and OXA fibers in the hamster and degu were moderate to low in the Pa and SO. Second, in the degu, a very dense network of OXA and OXB fibers was present in the VMH (Figure 10D), but in the other species fiber densities in this region were moderate to low. The OXA and OXB fiber complex in the degu VMH was the single most densely distributed network of orexin fibers in this animal. Third, in the hamster, OXA- and OXB-IR fibers in the lateral mammillary nucleus (LM) were fairly dense, while in the other three species fibers in the LM were sparse (Figure 11).

#### Thalamus

**Figure 12:**
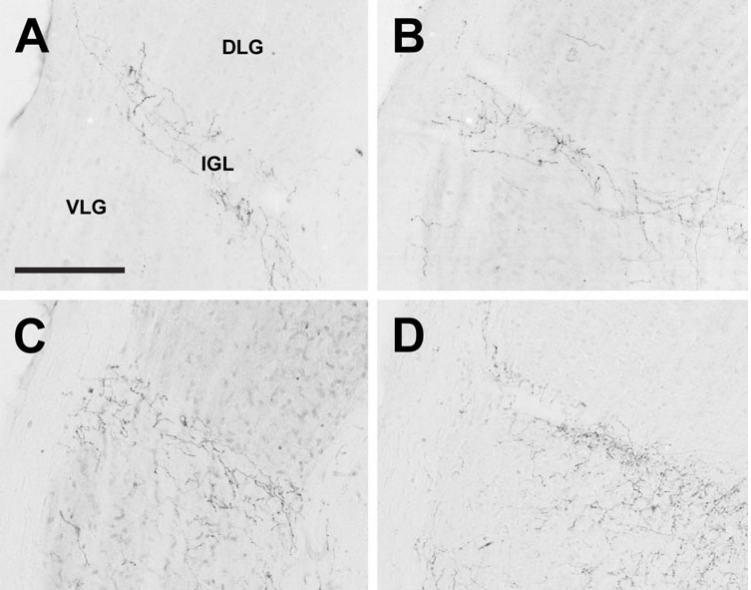
**Intergeniculate leaflet**. Photomicrographs of orexin A fibers in the intergeniculate leaflet (IGL) of the Long-Evans rat (A), grass rat (B), Syrian hamster (C), and degu (D). DLG: dorsolateral geniculate nucleus; VLG: ventrolateral geniculate nucleus. Scale bar = 200 μm.

**Figure 13:**
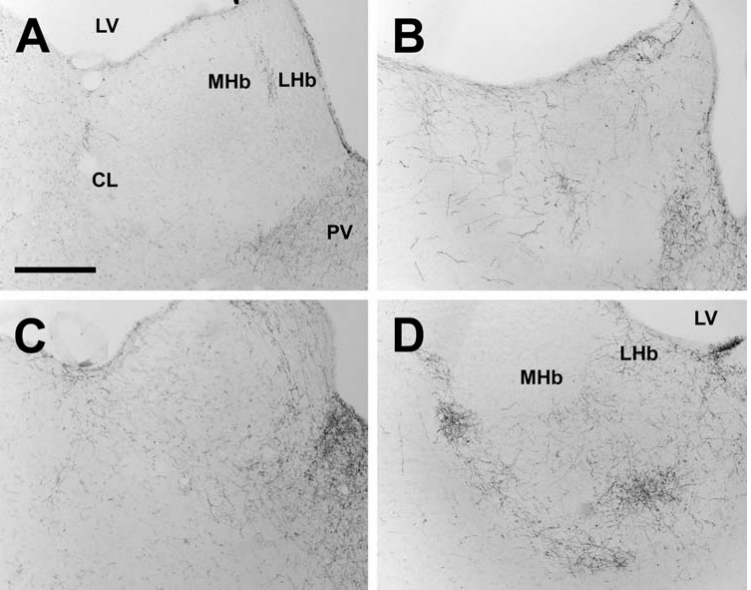
**Centrolateral nucleus**. Photomicrographs of orexin A fibers in the centrolateral nucleus (CL) of the Long-Evans rat (A), grass rat (B), Syrian hamster (C), and degu (D). Note higher density of orexin fibers in the degu CL in comparison with the other three species. LV: lateral ventricle; PV: paraventricular thalamic nucleus, MHb: medial habenular nucleus; LHb: lateral habenular nucleus. Scale bar = 300 μm.

**Figure 14:**
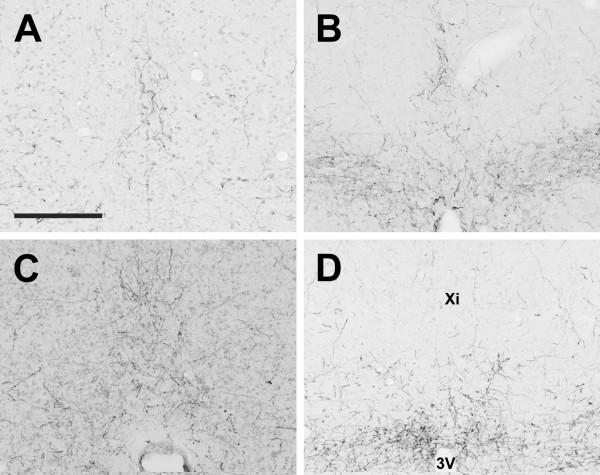
**Xiphoid nucleus**. Photomicrographs of orexin A fibers in the xiphoid nucleus (Xi) of the Long-Evans rat (A), grass rat (B), Syrian hamster (C), and degu (D). Note relative lack of orexin fibers in the degu Xi in comparison with the other three species. 3V: third ventricle. Scale bar = 200 μm.

In contrast to the dense networks of OXA and OXB fibers in the hypothalamus, orexin fibers were sparse or absent throughout much of the thalamus. The majority of orexin projections to the thalamus targeted midline structures, most notably the paraventricular thalamic nucleus (PV), which received dense or very dense orexin projections in all four species. In all species examined, projections of OXA or OXB fibers to lateral regions of the thalamus were most dense in the intergeniculate leaflet (IGL) (Figure 12); other lateral or ventral thalamic nuclei received little to no orexin input. Although the pattern of orexin innervation in the thalamus was fairly consistent, the degu differed from most or all of the other species examined in several regions. First, dense orexin fibers were present in the degu centrolateral (CL) and paracentral nuclei (PC) (Figure 13D). In the other three species, orexin fibers in these nuclei were mostly sparse or absent, although the LE rat did exhibit moderate innervation in the PC (Figure 13A). In contrast, the opposite pattern was observed in the xiphoid nucleus (Xi), which contained sparse fibers in the degu and dense orexin innervation in the other three species (Figure 14).

#### Telencephalon

**Figure 15:**
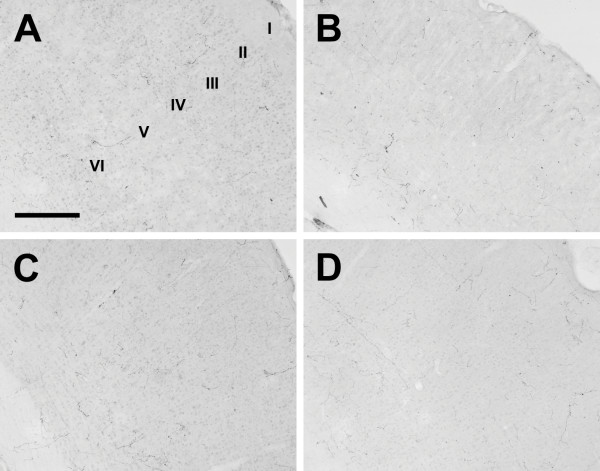
**Cerebral cortex**. Photomicrographs of orexin A fibers in the somatosensory cortex of the Long-Evans rat (A), grass rat (B), Syrian hamster (C), and degu (D). I-VI: Cortical layers 1–6. Scale bar = 300 μm.

**Figure 16:**
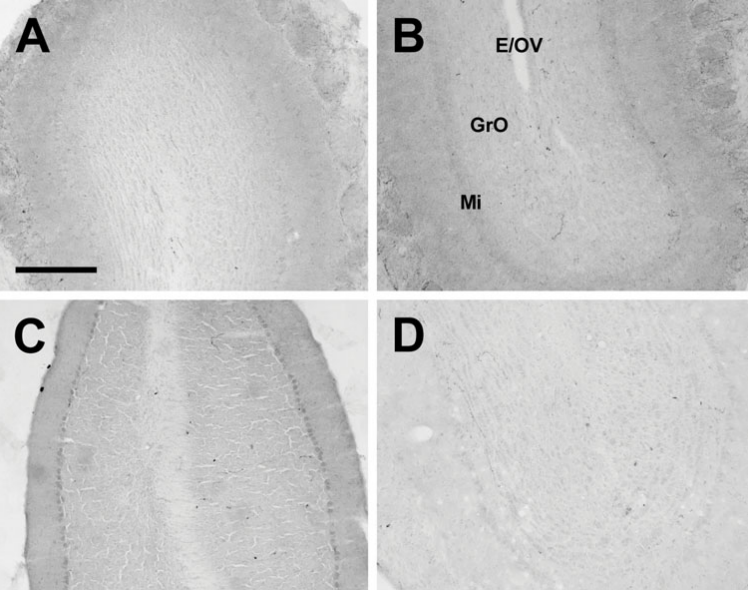
**Olfactory bulb**. Photomicrographs of orexin A fibers in the olfactory bulb of the Long-Evans rat (A), grass rat (B), Syrian hamster (C), and degu (D). E/OV: Ependymal layer/Olfactory ventricle; GrO: Granular cell layer; Mi: Mitral cell layer. Scale bar = 300 μm.

**Figure 17:**
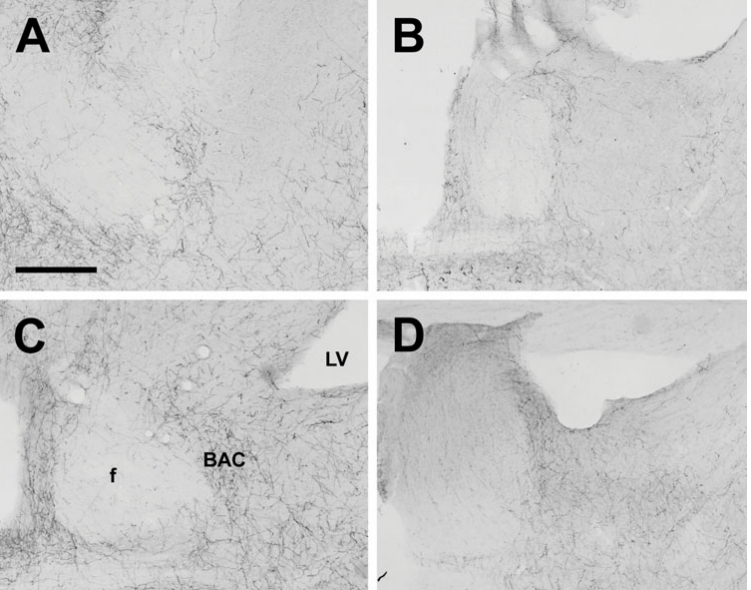
**Bed nucleus of the anterior commissure**. Photomicrographs of orexin A fibers in the bed nucleus of the anterior commissure (BNC) of the Long-Evans rat (A), grass rat (B), Syrian hamster (C), and degu (D). LV: lateral ventricle; f: fornix. Scale bar = 300 μm.

Outside of the diencephalon, the distribution of orexin-IR fibers was more heterogeneous (see Table 2). Orexin-A and OXB fibers were scattered throughout all areas of the cortex; these fibers were more concentrated in inner and outer cortical layers, and slightly reduced in Layers 3 and 4 in all animals (Figure 15). The olfactory bulbs exhibited no fibers in any species examined (Fig. 16), but sparsely scattered fibers were present in the olfactory cortex and tubercle in all species. Orexin fiber density was uniformly low to moderate in the septal nuclei, claustrum, and amygdala, with the exception of the hamster medial amygdaloid nucleus, where orexin fibers were more dense than in the grass rat and degu, but not significantly more so than in the LE rat. The density of both OXA and OXB fibers was very low in the hippocampus and basal ganglia in all animals. The bed nucleus of the anterior commissure exhibited moderate to dense orexin fiber innervation in all species (Fig. 17).

#### Midbrain

**Figure 18:**
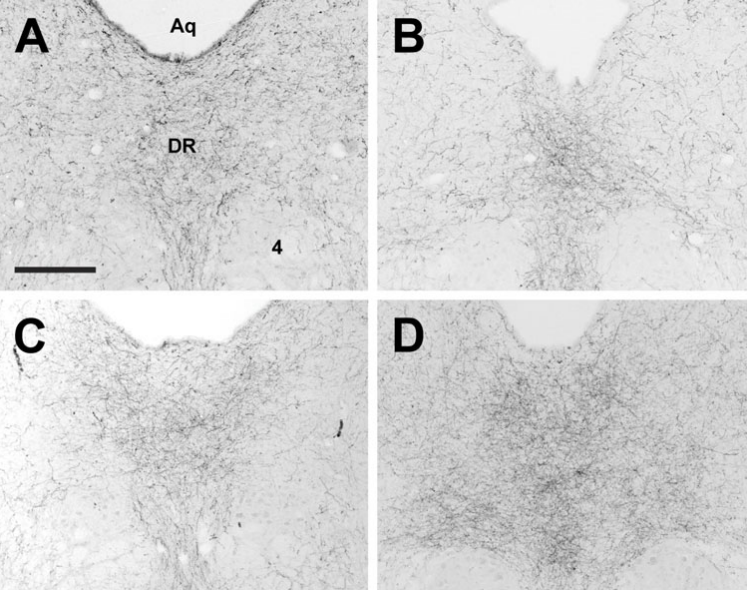
**Dorsal raphé nucleus**. Photomicrographs of orexin A fibers in the dorsal raphé nucleus (DR) of the Long-Evans rat (A), grass rat (B), Syrian hamster (C), and degu (D). 4: trochlear nucleus; Aq: cerebral aqueduct. Scale bar = 300 μm.

In general, OXA and OXB innervation of midbrain structures was moderate to low, with the exception of the dense fiber tracts in the periaqueductal gray and the very dense plexus of OXA and OXB fibers projecting to the raphé nuclei observed in all species (Figure 18).

#### Hindbrain

**Figure 19:**
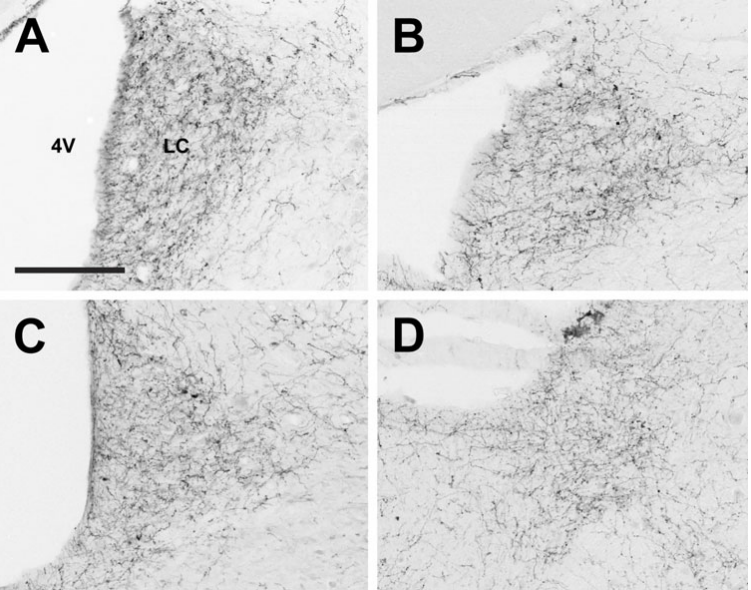
**Locus coeruleus**. Photomidcrographs of orexin A fibers in the locus coeruleus (LC) of the Long-Evans rat (A), grass rat (B), Syrian hamster (C), and degu (D). 4V: fourth ventricle. Scale bar = 200 μm.

In the hindbrain, OXA and OXB-IR fiber densities were moderate to low in the ascending brainstem tracts, motor and precerebellar nuclei, and were sparsely distributed or absent in the cochlear, vestibular, and olivary nuclei in all species. Visceral sensory nuclei, such as the nucleus of the solitary tract (Sol) and the Kölliker-Fuse nucleus, exhibited moderate to dense innervation by OXA and OXB fibers in all species, but the nucleus with the most extensive network of orexin fibers observed in the hindbrain was the LC (Figure 19). In the LE rat, grass rat, and hamster, the LC was the most densely innervated structure observed in the brain. In the degu, the network of OXA and OXB fibers present in the LC was quite dense, but was slightly less so than that observed in the VMH.

#### Cerebellum

**Figure 20:**
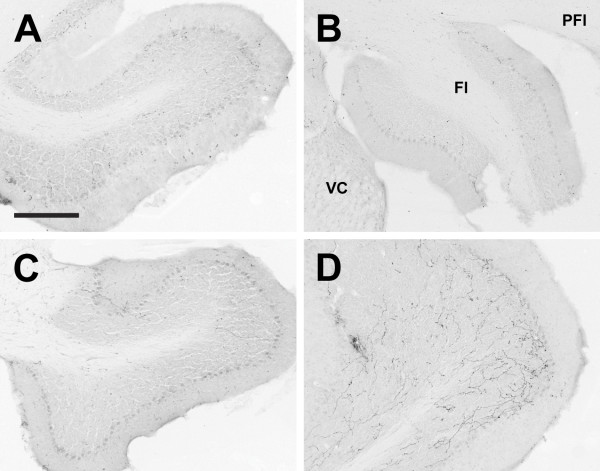
**Flocculus**. Photomicrographs of orexin A fibers in the flocculus (Fl) of the Long-Evans rat (A), grass rat (B), Syrian hamster (C), and degu (D). Note higher density of orexin fibers in the degu Fl in comparison with the other three species. PFl: paraflocculus; VC: ventral cochlear nucleus. Scale bar = 300 μm.

In the cerebellum, very few orexin fibers were observed in the LE rat, grass rat or hamster, but the degu was markedly different. In the degu cerebellum, moderate OXA and OXB fiber densities were observed in some deep cerebellar nuclei, as well as the flocculus (Figure 20D).

#### Circumventricular regions and ependyma

In circumventricular organs, OXA innervation in all animals was moderate to low, and there were even fewer OXB fibers. In the LE rat and grass rat, the median eminence exhibited greater densities of OXA-IR fibers relative to the other two species, although this difference is likely explained by axonal projections from the OXA-IR cells in the Pa and SO in these two species.

## Discussion

### General observations

We present here a thorough examination of the distribution of OXA- and OXB-IR cell bodies and fibers in four species. Prior to this study, no published data were available describing orexin A or B in the LE rat strain or in the degu, and no data were available for OXA in the grass rat or OXB in the Syrian hamster. The data presented here correspond well with previously published examinations of OXA in the Syrian hamster [25,27] and of OXB in the grass rat [48].

At the most general level, the three most significant observations in this study were: (1) There is a high degree of correspondence within species between OXA and OXB; (2) The overall pattern of orexin cell and fiber distribution is very similar in these four species and to those described previously for other strains and species of mammal, including Wistar and Sprague-Dawley rats [19-22,24], Djungarian hamsters [26,27] and primates [28,30,85]; and (3) Despite the overall similarity between species, there are several striking differences, both with respect to the distribution of orexin-IR cells, as well as in the relative density of orexin-IR fibers in some regions.

The implications of the observation that there are high levels of correspondence between OXA and OXB distribution within species are twofold. First, although at least some OXA-only cell types were identified in the current study (see below), our data support the general assumption that the majority of cells and fibers expressing OXA immunoreactivity also express OXB, consistent with previous reports [40]. Within each species, the PCA analysis showed nearly identical values for both OXA and OXB (Table 2), suggesting that both the pattern of distribution and region-specific density of OXA and OXB fibers are largely identical within each individual. Second, the overlap between OXA- and OXB-IR fibers in all species studied suggests that the individual actions of OXA and OXB in regulation of physiology and behavior are more likely due to differences in the distribution of the orexin receptor subtypes within the brain than to differential distribution of the two forms of the peptide. The two orexin receptor subtypes do exhibit marked differences in distribution in rats [33-35], and both rat and human orexin receptors express differential responsiveness to OXA or OXB [1,31].

The second major pattern seen in this study, that species are very similar with respect to the distribution of orexin fibers and cells, suggests that orexin networks are strongly conserved. This implies that the functions of orexin are likely to be similar in the LE rat, grass rat, Syrian hamster, and degu, as well as in other species. In addition, the PCA analysis appears to show stronger correlations between species which are more closely related; for example, correlations between the LE rat and grass rat are stronger than those between the LE rat and degu (Table 2). This finding suggests that the differences observed between species may be due to differences in phylogenetic history rather than differences in the function of the orexins in nocturnal and diurnal animals. The orexins are strongly conserved peptides [86-88]. The orexin gene arose early in chordate evolution [89], and has been retained in all major vertebrate classes, including fish [90,91], amphibians [87,92,93], reptiles [94], birds [88], and mammals [86]. This pattern is evidence for an essential functional role for the orexins in mediation of behavioral and physiological processes common to all vertebrates, and further implies strong evolutionary constraints against modification of projections from orexin cells. This conclusion is consistent with the emerging consensus that the orexins are important in stabilizing transitions between the sleeping and waking states, rather than playing an important role in establishing sleep-wake patterns [95].

Lastly, though there are relatively few major differences among species in the distributions of these highly conserved peptides, their presence suggests functional differences related to the life history, behavior, or physiological organization of different animals. We focus the rest of our discussion on some of the differences among species with respect to in the distribution of orexin-IR cells and fibers.

### Species differences in OXA and OXB cell distribution

Though few in number, the three differences observed between species in the overall distribution of orexin-IR cell bodies were striking. First, orexin cells were conspicuously absent in the hamster PeF. Second, the distribution of the main body of orexin-IR perikarya within the caudal diencephalon in the degu was markedly different from that seen in the other species examined in this study. Third, the presence of OXA-IR cell bodies in the Pa and SO was noted in three of the four species examined.

In the hamster, previous reports stated that orexin-IR neurons were primarily located in LHA and PeF [25,27]. In the present study, orexin-IR cells were largely absent in the PeF, and were primarily found in the LHA dorsal to the perifornical region (Figure 7). It is possible that the difference observed between the present data and those described in previous reports reflect differences in delineation of specific hypothalamic areas. The present study relied on a hamster-specific atlas to identify brain regions [82]; as noted by the authors of this atlas, the hamster brain is in many respects organized quite differently from that of the rat [82]. At least one previous study [27] used a rat brain atlas [81]; the second report [25] did not indicate the method used for identification of different regions. In comparison with the rat brain atlas, the region identified as PeF in the hamster atlas is much smaller and situated closer to the fornix [81,82]. However, even if an expanded rat-like definition of the PeF is applied to the hamster, it is clear that the number of orexin neurons observed near the fornix is lower in the hamster than in the other three species examined in this study (see Figures 5, 6, 7, 8).

In the degu, two main differences were noted in the distribution of orexin-IR perikarya within the caudal diencephalon in comparison with the other three species. First, the degu exhibited orexin neurons through a much larger extent of the rostral-caudal axis of the hypothalamus, with cells expressing OXA or OXB extending from the RCh to the SuM (Figure 8). Second, the overall organization of the main body of orexin neurons differed in the degu relative to the other species in that two distinct clusters of orexin-IR cells were present, one dorsomedial to the fornix, and a second group ventrolateral to the fornix. In the caudal portion of their distributions, these groups merged into a single scattered group of orexin-IR cells near the midline, below the third ventricle. The differences in the overall distribution of orexin neurons in the degu relative to the other three species raise the possibility that orexin cells in the ventrolateral LHA, TC, and SuM of the degu receive different inputs or project to different targets than orexin cells in the dorsal LHA and PeF of the rat, grass rat, or hamster. Projections from these neurons might contribute to the overall differences in orexin fiber distribution in the degu relative to the other three species (see below).

Although the main body of orexin neurons in the LE rat, grass rat and hamster LHA were fairly consistent with those described previously, the presence of OXA-IR perikarya in the magnocellular nuclei of the hypothalamus in these three species has not been described previously. The presence of orexin-IR neurons outside of the lateral hypothalamus is not without precedent. Earlier reports suggested the presence of OXA-IR neurons in the median eminence [21], and OXB-IR neurons in limbic structures [46]. In general, the OXA-IR perikarya in the Pa, SO and SOR were not as darkly stained as were OXA-IR neurons in the LHA and PeF, and the number of OXA-IR cells in these nuclei varied between species (Figure 2, Figure 3). Orexin A-IR cells in the Pa and SO were fairly dense in the grass rat, with fewer OXA-IR cells visible in the LE rat Pa and SO. In the hamster, immunoreactivity for OXA was generally negligible in cells of the Pa, but a small number of well-defined OXA-IR neurons were visible in the SO.

The magnocellular neurosecretory cells of the Pa and SO are primarily known for the production of arginine vasopressin (AVP) and oxytocin (OT), peptide hormones delivered to the pituitary via projections to the median eminence [reviewed in [96]]. These cells have been studied extensively, and the normal functions of these nuclei are well-understood. The presence of a previously unreported peptide in these cells is therefore quite surprising. Several lines of evidence suggest that the OXA immunoreactivity in these nuclei reflects the presence of the peptide rather than non-specific immunoreactivity. First, tissue that has been blocked by preabsorption of the OXA antibody with OXA blocking peptide reduces or eliminates the staining of cell bodies in the Pa, SO, and LHA of all species examined (Figure 4). Second, as immunohistochemical procedures for both OXA and OXB were identical except for the primary antibody used, nonspecific binding of the secondary antibody is unlikely (see Methods). Third, the OXA immunoreactivity seen in the Pa, SO, and SOR does not appear to be the result of the primary antibody binding to an orexin-like peptide that is normally found in these magnocellular nuclei, as tissue reacted with the same antibodies does not show any evidence of orexin-IR perikarya in the Pa or SO of the degu (present study), Sprague-Dawley rat (personal observation), or the golden-mantled ground squirrel (*Spermophilus lateralis*) (personal observation). Finally, the OXA-IR neurons in the SO and Pa are not the result of the antibody binding to AVP, as other regions known to produce AVP, such as the SCN, do not exhibit similar immunoreactivity (see Figure 9). However, as the presence of prepro-orexin mRNA has not yet been shown in these nuclei, it is important to note that the OXA-IR cell bodies visible in the SO and Pa could be due to nonspecific binding of the antibodies used in this study. It is also possible that these neurons do not actually produce OXA, but instead may selectively uptake and store OXA produced by cells elsewhere in the hypothalamus.

The Pa and SO are involved in a number of systems in the brain. These nuclei project to the pituitary via the median eminence, where they release vasopressin and oxytocin, peptides involved in reproduction, regulation of blood pressure, and control of the hypothalamic-pituitary-adrenal (HPA) axis [reviewed in [97]]. The presence of OXA in these magnocellular nuclei in the LE rat, but not in the inbred Wistar rat, might be related to the stress response, which is higher in the LE rat than in the Wistar rat [98]. Orexin A actions in the Pa appear to potentiate the stress response, in part by modulating the release of corticotrophin releasing factor (CRF) [99-101]. In contrast, OXB appears to have a suppressive rather than a stimulatory effect on CRF release [102], raising the possibility that differences in OXA distribution might contribute to differences in stress-related behavior and physiology between different strains of rats.

### Species differences in OXA and OXB fiber distribution

There were several specific regions in which one or two species tended to strongly diverge from the rest in terms of orexin-IR fiber density. In two of these regions -the magnocellular neurosecretory nuclei of the hypothalamus in the LE rat and grass rat, and the PeF in the hamster – differences in OXA and OXB fiber density are directly related to the presence of OXA cell bodies or the absence of any orexin-IR neurons, respectively. A third area, the median eminence in the LE rat and grass rat, is also likely different with respect to OXA fiber density than in the hamster and degu because of projections originating from OXA-IR cells in the LE rat and grass rat Pa and SO (although it is worth mentioning that the hamster, which also exhibited OXA neurons in the SO, did not differ from the degu with respect to OXA fiber density in this region). The species differences in relative fiber density in other brain regions raise more interesting questions. We will focus the remainder of this discussion on the brain regions within each species that showed the most unique patterns of innervation – specifically, the LM of the hamster, and the VMH, CL, Xi, and cerebellum of the degu.

Orexin fiber density in the lateral mammillary nucleus was much higher in the hamster than in the other three species, in which orexin fibers were sparse or absent in this region (Figure 11). The mammillary nuclei in general receive dense input from the hippocampus [reviewed in [103]], and lesion studies suggest a role for the LM in spatial learning and memory in rats [104]. However, it is not obvious at this time why the hamster LM should exhibit increased orexin fiber input, which is presumably associated with arousal, relative to the other species. It should be noted that hamsters were kept in a 14:10 LD cycle in order to prevent testicular regression, which is known to affect orexin cells in some species [47]. Therefore, although all species in the current study were in the same reproductive condition, the hamsters were maintained in a slightly different photoperiod than the other species. It is theoretically possible that the longer daylengths to which the hamsters were exposed are responsible for the relatively high density of orexin fibers in their LM. We view this as unlikely, as most photoperiodic effects on behavior and physiology are mediated by changes in gonadal function, and there is no evidence of gonad-independent effects of daylength on the orexin system.

The most striking differences in orexin fiber distribution in this study were seen in the degu. In this species, orexin fiber density was lower in the xiphoid nucleus (Figure 14D) and much higher in the centrolateral nucleus of the thalamus (Figure 13D) in comparison to the other three species. The degu also exhibited an extremely dense network of orexin-IR fibers in the VMH, predominantly in the ventromedial portion of this nucleus (Figure 10D). In the other species, orexin fiber density in the VMH was moderate to low, while this nucleus represented the region of greatest fiber density observed in the degu. With respect to the cerebellum, the degu exhibited moderately dense orexin-IR innervation of the cerebellar nuclei and flocculus, while in the other three species these regions were sparsely innervated at best (Figure 20). The grass rat, LE rat, and Syrian hamster are relatively closely related to each other (all are members of the Suborder Sciurognathi, Family Muridae), and the degu (Suborder Hystricognathi, Family Octodontidae) is relatively distantly related to them [68]. It is therefore possible that the differences in fiber distribution observed between the degu and the other three species are a reflection of phylogenetic history and constrains associated with it, rather than functional differences among these rodents. However, it is tempting to speculate on the function of orexin projections to these regions in the degu.

Although the implication of the lack of orexin input to the xiphoid nucleus is unclear, the networks of orexin-IR fibers in the degu CL, VMH and cerebellum could all play a role in anti-predator behavior during periods of heightened arousal, such as sentinel activity. When feeding, degus alternate sentinel duties and produce alarm calls to warn conspecifics of danger [reviewed in [69]]. Sentinel duty in the degu requires that an animal both detect potential threats, and respond properly to threats once identified. The orexin-IR fibers in the flocculus might aid in the visual detection of threats. Predator avoidance behavior in the degu appears to be based primarily upon visual scans for aerial or ground-level threats [105]. Pursuit of a visually acquired target requires smooth, coordinated eye movements to maintain visual contact with a constantly moving stimulus [106]. The flocculus has been shown to be important in maintaining such predictive eye movements during visual tracking [107]. The CL and the VMH may be involved in appropriate response to predators. Amygdalar projections from the CL and other intralaminar thalamic nuclei are thought to engage fear responses in rats [reviewed in [108]], and the CL specifically appears to be important in cognitive awareness and executive decision making [109]. The VMH is part of the cerebral-hypothalamic "behavior control column" mediating somatomotor aspects of complex motivated behavior [110]. The dorsomedial region of this nucleus is specifically implicated in predator-induced defensive responses [111]. The three brain regions in the degu receiving heavier than average orexin innervation may all be involved in anti-predator behaviors. It is possible that coordination and activation of these systems during periods of heightened arousal may be an important function of orexins in the degu.

## Conclusion

In summary, with respect to the overall distribution of orexin, this study confirms the high degree of similarity among species overall, but also reveals some significant differences. The distribution of orexin-IR cell bodies and fibers is quite similar among the Long-Evans rat, grass rat, hamster and degu, and is largely consistent with previously published reports [19-22,24,25,27,48]. With respect to orexin fibers, the high levels of congruence between species suggest a strongly conserved common role for OXA and OXB in the maintenance of arousal state, modulation of somatomotor activity, and control of ingestive behavior. On the other hand, there are some significant species differences in the distribution of orexin cell bodies as well as the density of orexin-IR fibers in some regions. With respect to cell bodies, the present study describes specific species differences in the organization of the main body of OXA- and OXB-IR neurons in the lateral hypothalamus, and also provides evidence for a previously undescribed population of OXA-IR neurons in the magnocellular neurosecretory nuclei of the hypothalamus. These differences in orexin cell distribution raise the possibility that some subpopulations of orexin-IR neurons might differ with respect to afferent inputs, suggesting potential differences in activation of orexin neurons among species. In addition, while the overall distribution and relative densities of orexin fibers are quite similar across species, there are striking differences in some regions. These presumably reflect interspecific variability in the importance or function of acute orexin actions in specific nuclei, and suggest that we should not assume that the system operates identically across species.

## Abbreviations

Arcuate nucleus (Arc)

Arginine vasopressin (AVP)

Bed nucleus of the anterior commissure (BAC)

Bed nucleus of the stria terminalis (BNST)

Centrolateral thalamic nucleus (CL)

Cerebral aqueduct (aq)

Corticotrophin releasing factor (CRF)

Diaminobenzidine (DAB)

Dorsal hypothalamic area (DA)

Dorsolateral geniculate nucleus (DLG)

Dorsomedial hypothalamic nucleus (DMH)

Ependymal layer (E)

Flocculus (Fl)

Fornix (f)

Fourth ventricle (4V)

Granular cell layer of olfactory bulb (GrO)

Hypothalamic-pituitary-adrenal axis (HPA)

Immunoreactive (IR)

Intergeniculate leaflet (IGL)

Lateral hypothalamic area (LH)

Lateral habenular nucleus (LHb)

Lateral mammillary nucleus (LM)

Lateral ventricle (LV)

Leuteinizing hormone releasing hormone (LHRH)

Light-dark (LD)

Locus coeruleus (LC)

Long-Evans (LE)

Mammillothalamic tract (mt)

Medial habenular nucleus (MHb)

Medial mammillary nucleus (MM)

Mitral cell layer of olfactory bulb (Mi)

Neuropeptide-Y (NPY)

Normal donkey serum (NDS)

Nucleus of the solitary tract (Sol)

Olfactory ventricle (OV)

Optic chiasm (OC)

Orexin A (OXA)

Orexin B (OXB)

Orexin receptor 1 (OX_1_R)

Orexin receptor 2 (OX_2_R)

Oxytocin (OT)

Paracentral thalamic nucleus (PC)

Paraventricular hypothalamic nucleus (Pa)

Paraventricular thalamic nucleus (PV)

PBS with 0.3% Triton-X 100 (PBS-TX)

Paraflocculus (PFl)

Perifornical region (PeF)

Phosphate-buffered saline (PBS)

Posterior hypothalamic area (PH)

Principal components factor analysis (PCA)

Retrochiasmatic area (RCh)

Sprague-Dawley (SD)

Subincertal thalamic nucleus (SubI)

Suprachiasmatic nucleus (SCN)

Supramammillary nucleus (SuM)

Supraoptic nucleus (SO)

Supraoptic retrochiasmatic nucleus (SOR)

Third ventricle (3V)

Trochlear nucleus (4)

Tuberum cinerum (TC)

Ventral cochlear nucleus (VC)

Ventrolateral geniculate nucleus (VLG)

Ventromedial hypothalamic nucleus (VMH)

Xiphoid nucleus (Xi)

## Competing interests

The author(s) declare that they have no competing interests.

## Authors' contributions

JN conceived of the study, carried out the data collection and analysis, and drafted the manuscript. LS participated in the design of the study, assisted in interpretation of the data, and helped draft the manuscript.
